# Technical comment on Condamine et al. (2019): a cautionary note for users of linear diversification dependencies

**DOI:** 10.1111/ele.13483

**Published:** 2020-03-25

**Authors:** Alexander Gamisch

**Affiliations:** ^1^ Department of Biosciences University of Salzburg Hellbrunnerstrasse 34 5020 Salzburg Austria

**Keywords:** Absolute values, dependency, diversification, linear, misinterpretation, negative rates, RPANDA

## Abstract

Condamine et al. (2019; 22: 1900–1912) fitted linear and exponential functions of time‐dependent, diversity‐dependent and temperature‐dependent diversification to investigate diversification dynamics of tetrapod families. Here I highlight potential misinterpretations when using linear diversification dependencies and provide some clarifications.

## Comment

Condamine *et al. *(2019) investigated diversity‐dependent and temperature‐dependent phylogenetic models of diversification across 218 tetrapod families, along with constant‐rate and time‐dependent models. This technical comment pertains to the linear time‐dependent model, which can lead to unintended model fits under certain circumstances and may therefore only used with caution in model testing.

**Figure 1:**
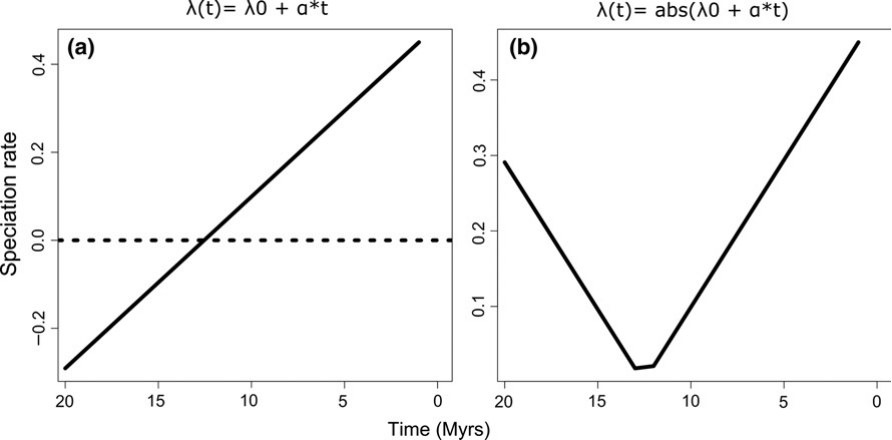
Speciation rate through time based on the parameters of the Rhinolophidae clade (λ0 = 0.45, α = −0.039, clade age (t) = 19.6 in million years (Myrs); see Table 1) for two linear time‐dependent diversification functions: (a) the linear function λ(t) = λ0 + α*t, as described in the Condamine et al. (2019) study and the RPANDA documentation; (b) the function λ(t) = abs(λ0 + α*t), as effectively used in the RPANDA computation. Note, the rate in a) crosses zero (stippled line) and then becomes negative, whereas the rate in (b) first decreases backwards and then increases again (near parabolic).

The authors used the *fit_bd* function of the R‐package RPANDA (Morlon *et al.* 2016) to fit birth–death models to reconstructed phylogenies, in which speciation rates λ and/or extinction rates µ are varied as functions of time t (backwards in time from the present to the past). The authors (p.1902) took …‘this function to be either linear λ(t) = λ0 + α*t and/or µ(t) = µ0 + β*t or exponential λ(t)= λ0 * e^α*t^ and/or µ(t)= µ0 * e^β*t^ where λ0 (β0) is the speciation (extinction) rate at present and α (β) measures the sign and rapidity of the time variation. A positive α (β) reflects a slowdown of speciation (extinction) towards the present, whereas a negative α (β) reflects a speed‐up of speciation (extinction) towards the present….’ This is problematic because, under the linear function λ(t) = λ0 + α*t, a negative change factor (α) leads to negative speciation rates once α*t is more negative than λ0 is positive or T (crown age)> abs(λ0/α) (the same for µ0 and β; Fig. 1a). While negative net diversification rates (*r* = λ–µ) are plausible under certain circumstances (e.g. evolutionary dead ends; Freyman & Höhna 2019 and references therein), negative speciation or extinction rates make little sense biologically. For this latter reason, the RPANDA *fit_bd* function uses absolute values for the computation, no matter what kind of function is specified (H. Morlon, personal communication). This subtle but important aspect is currently only visible in the code of the *fit_bd* function but not explicitly mentioned in text or the documentation of RPANDA v.1.7 (Morlon *et al.* 2016, 2020), and therefore likely escapes the attention of most users. In other words, even if the user specifies a linear time dependency of λ(t) = λ0 + α*t (e.g. in the speciation function f.lamb), it is the equation λ(t) = **abs**(λ0 + α*t) that is effectively used during computation. This, however, has noteworthy implications for all RPANDA (*fit_bd*) phylogenies/model fits where a negative change factor and T> abs(λ0/α) would allow for negative rates during the history of the clade according to the linear function λ(t) = λ0 + α*t (Fig. 1a). This special case applies to c. 9% (20/218) of the best fitting models of the tetrapod phylogenies used in the Condamine *et al. *(2019) study (Tab. 1). In fact, for those phylogenies, a rate curve is fitted that is decreasing backwards in time until λ0 = α*t and, from that time backwards, the rate is increasing again (Fig. 1b). Under those conditions, users of RPANDA need to be aware (i) that it is no longer a ‘true’ linear function (as strictly defined by λ(t) = λ0 + α*t) that is fitted and (ii) that the sentence (p.1902) …’*A positive α (β) reflects a slowdown of speciation (extinction) towards the present, whereas a negative α (β) reflects a speed‐up of speciation (extinction) towards the present…’.* is no longer accurate. Although the above problem was mainly observed with linear time‐dependent diversification models, it could also be important for the RPANDA environmental‐dependent model (*fit_env* function) of diversification (e.g. when a linear dependency of λ or µ with the environmental variable would lead to negative λ or µ rates during the history of a clade).

**Table 1:** The 20 out of 218 tetrapod phylogenies (taken from Table S4 of Condamine *et al.* 2019) for which the best fitting model implies a linear time dependency that, in turn, would lead to negative diversification rates (either λ or μ) or backwards decreasing and increasing rates when the model parameter values are inserted in the functions λ(t) = λ0 + α*t or λ(t) = **abs**(λ0 + α*t) (the same for µ0 and β) respectively (see also Fig. 1a and b). Clade age (t) is in million years. For model descriptions and acronyms see Table 1 of Condamine *et al. *(2019)

Clade	Best fitting model	Clade age (t)	λ0	α	μ0	β
Amphibia:Bombinatoridae	BTimeVar_LIN*	87.3	0.043	−0.001	−	−
Amphibia:Eleutherodactylidae	BTimeVarDTimeVar_LIN†	62.7	0.018	0.008	0.07	−0.009
Amphibia:Hynobiidae	BTimeVarDTimeVar_LIN†	134.7	0.007	0.003	0.042	−0.003
Amphibia:Plethodontidae	BTimeVarDTimeVar_LIN†	103.4	0.021	0.006	0.025	−0.005
Amphibia:Ranidae	BTimeVarDTimeVar_LIN†	88.7	0.001	0.006	0.041	−0.004
Aves:Anatidae	BTimeVarDCST_LIN‡	97.9	0.421	−0.007	0.233	–
Aves:Cettiidae	BTimeVar_LIN*	33.9	0.177	−0.007	–	–
Aves:Galbulidae	BTimeVar_LIN*	41.8	0.105	−0.003	–	–
Aves:Nectariniidae	BCSTDTimeVar_LIN§	41.1	0.209	–	0.261	–0.016
Aves:Picidae	BTimeVarDTimeVar_LIN†	77.5	0.163	0.037	0.142	–0.055
Aves:Rallidae	BTimeVar_LIN*	70.4	0.138	–0.002	–	–
Aves:Thraupidae	BTimeVarDTimeVar_LIN†	19	0.259	0.049	0.432	–0.092
Aves:Turdidae	BTimeVarDTimeVar_LIN†	31.7	0.041	0.022	0.094	–0.025
Mammalia:Cebidae	BTimeVarDTimeVar_LIN†	19.2	0.101	0.374	0.145	–0.352
Mammalia:Leporidae	BTimeVarDTimeVar_LIN†	29.8	0.062	0.124	0.16	–0.109
Mammalia:Rhinolophidae	BTimeVar_LIN*	19.6	0.45	−0.039	–	–
Mammalia:Soricidae	BTimeVarDTimeVar_LIN†	46.6	0.07	0.023	0.04	−0.026
Mammalia:Spalacidae	BTimeVar_LIN*	48	0.108	−0.003	–	–
Mammalia:Vespertilionidae	BTimeVarDTimeVar_LIN†	51.5	0.058	0.028	0.1	−0.034
Squamata:Colubridae	BTimeVarDTimeVar_LIN†	52.4	0.008	0.014	0.032	−0.01

* BTimeVar_LIN: Speciation linear variable with time and no extinction.

† BTimeVarDTimeVar_LIN: Both speciation and extinction linear variable with time.

‡ BTimeVarDCST_LIN: Speciation linear variable with time and constant extinction.

§ BCSTDTimeVar_LIN: Constant speciation and extinction linear variable with time.

This comment is in no way intended to discredit the results of the Condamine *et al. *(2019) study. Rather, it should raise awareness of unintended implications of the linear‐dependent diversification models that could generate negative rates (Fig. 1a) that would be positivised in the current implementation of RPANDA (Fig. 1b). One possible alternative could be to specify a function that can vary its form when rates change (e.g. Morlon *et al.* 2011). In any event, I recommend users to critically examine whether the models fitted to their data are realistic or meaningful (e.g. by filling in the parameter values in the function specified and using the *plot_fit_bd* or *plot_fit_env* function). Alternatively, users who find these subtleties confusing may choose to avoid the linear functions currently implemented in RPANDA (*fit_bd, fit_env*) altogether and instead use the program’s exponential functions, which sidestep all the above problems entirely.

## Authorship

AG designed the study, has analysed the data and has written the manuscript.
